# In Vitro Antioxidant and Antimicrobial Activities of* Ephedra gerardiana* (Root and Stem) Crude Extract and Fractions

**DOI:** 10.1155/2017/4040254

**Published:** 2017-04-12

**Authors:** Aman Khan, Gul Jan, Afsar Khan, Farzana Gul Jan, Ali Bahadur, Muhammad Danish

**Affiliations:** ^1^Department of Botany, Hazara University, Mansehra, Pakistan; ^2^School of Life Science, Lanzhou University, Lanzhou, China; ^3^Department of Botany, Abdul Wali Khan University, Mardan, Pakistan; ^4^Department of Chemistry, COMSATS Institute of Information Technology, Abbottabad 22060, Pakistan

## Abstract

The utilization of medicinal plants to treat infectious disease is a common practice in developing countries worldwide. The present study was aimed at evaluating the crude extracts of* Ephedra gerardiana* (root and stem) with different chemicals for antioxidant and antimicrobial (fungal and bacterial) potential. The results revealed that the ethyl acetate fractions of* E. gerardiana* (root and stem) have significant free radical scavenging potential with values 2.96 ± 0.39 and 2.73 ± 0.84 while *n*-butanol and aqueous fractions showed IC_50_2.69 ± 0.26 and 3.44 ± 0.69 *µ*g/ml in stem. Furthermore, crude extract and fractions also revealed promising antibacterial activities against all tested microbial strains while aqueous fraction showed no activities against* Bacillus subtilis*,* Kleibsiella pneumoniae*, and* Pseudomonas aeruginosa*. Interestingly, all crude extracts and fractions were nonactive against fungal strain,* Aspergillus niger* and* Aspergillus flavus*, as compare to control. In summary, the* Ephedra gerardiana* (root and stem) extract and fraction possess antioxidant activities, which might be helpful in preventing or slowing the progress of various oxidative stresses, suggested to be a strong pharmaceutical agent.

## 1. Introduction

The northern areas of Pakistan are considered to be rich in biodiversity and contain more than 400 species of medicinal plant species including genus ephedra, which is evergreen gymnosperm, belonging to family Ephedraceae [1]. Ma-huang is the drug isolated from* Ephedra,* used in Chinese medicine for 5000 year to cure various human diseases such as fever, nasal congestion, and asthma [2]. In Japan, ephedra genus is used at a rate of some 300 tons a year due to their addition as a crude drug in several Sino-Japanese medicine; preparations have been widely used as a treatment for fever, nasal congestion, and asthma [3]. Furthermore, the ephedra genus contains bronchial dilator, ephedrine, and other ephedrine alkaloids. It has been also used for many years in traditional medicine to treat allergies, bronchial asthma, chills, colds, coughs, edema, fever, flu, headaches, and nasal congestion and has been a natural source of alkaloids such as ephedrine, pseudoephedrine, pseudoephedrine, and other related compounds [4]. Similarly, the extracts with various chemicals from medicinal plants have been tested and showed the effectiveness of traditional herbs against microorganisms; as a result plants are one of the bedrocks for modern medicine to attain new principles [5]. Thus, medicinal plants can offer a wealth for their biological activities, such as antimicrobial, antioxidants, antimalarial, and anticancer activities.

Therefore, the exploration of plants needs attention to isolate compounds that can act as suitable antioxidants and antimicrobial agent instead of synthetic compounds [6]. Recently, the natural antioxidants have significantly increased in food and cosmetic and therapeutic products, since they have multifacetedness in their mass and amount of activities and provide huge scope in modifying imbalance [7]. Similarly, the aqueous extract of the* Ephedra aphylla* is used as strong inhibiter against malarial parasitic protozoan,* plasmodium falciparum* [8], and the compounds which were isolated from* Ephedra aphylla* have no estrogenic activity but most of the Lignans exhibited moderate antioxidant activity without any cytotoxicity. Up to now, some species of genus ephedra were screened for antimicrobial activities including,* E. altissima* [9],* E. transitoria* [10],* E. breana* [11], and* E. gerardiana* (leaf) [12], while, as for their antioxidant potential,* E. laristanica, E. sarcocarp* [13], and* E. gerardiana* (ethanol extract) [14] were investigated. In the light of this previous study, we sought to evaluate the crude extracts and fractions of* E. gerardiana* (root and stem) for free radical scavenging potential and antimicrobial (fungal and bacterial) activities.

## 2. Materials and Methods

### 2.1. Plant Material

**Figure 1:**
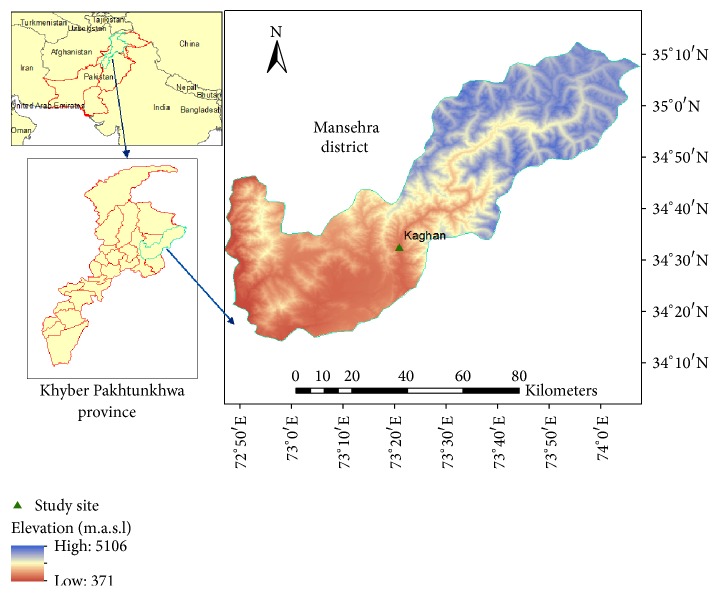
Study area, the plant was collected from area (Δ) as shown in map.

*Ephedra gerardiana* was collected from the Kaghan valley of Khyber Pakhtunkhwa, Pakistan (Figure 1), and plant specimen was deposited with the voucher number 3550 in herbarium of Hazara University, Mansehra, Pakistan.

### 2.2. Extraction and Fraction

Collected plant materials (root and stem) were dried at room temperature and grinded into fine powder. The powdered plant materials were soaked in methanol (commercial grade) for 12 days three times. The extract was filtered using filter paper and residue plant materials were soaked again in methanol and solvent was vaporized at 40°C through rotary evaporator [15]. The process was repeated three times and the methanol crude extract was obtained. The crude methanolic extract was suspended in distilled water with constant stirring and fractionated with *n*-hexane, chloroform (CHCl_3_), ethyl acetate (EtOAc), and n-butanol, respectively [16]. The solvents were evaporated at 40°C through rotary evaporator and different fractionations were obtained.

### 2.3. Chemicals

Propyl gallate (PG), 1,1-diphenyl-2-picrylhydrazyl (DPPH), 3-tert-butyl-4-hydroxyanisole (TBH), dimethyl sulfoxide (DMSO), sabouraud dextrose agar (SDA), nutrient agar, and nutrient broth. All chemicals and reagents were purchased from Merck (Germany) and Sigma-Aldrich (USA).

### 2.4. Stock Solution Preparation

Methanolic crude extracts and different fractions (*n*-hexane, chloroform, ethyl acetate, and *n*-butanol) of the plants were dissolved in dimethyl sulfoxide (DMSO) with the concentration of 1 mg/ml being evaluated for biological potential [17].

### 2.5. Antioxidant Activity (DPPH Assay)

Plants extracts were examined for their antioxidant activity on the basis of scavenging effects using stable 1,1-diphenyl-2-picrylhydrazyl (DPPH) method [18]; freshly DPPH solution was prepared and kept in the dark at 4°C. 1,1-Diphenyl-2-picrylhydrazyl (DPPH) (3.2 *µ*l) was dissolved in 25 ml methanol with steering regularly up to 30 minutes. Methanolic solutions of DPPH (90 *µ*l) were added to 10 *µ*l of plants extracts solution with different concentration. The mixture was incubated for 30 minutes at 37°C at room temperature; after incubation the absorbance was measured at 490 nm using multiplate reader (Bio-Tek Elx800™, Instruments, Inc., USA). Propyl gallate (PG) and 3-tert-butyl-4-hydroxyanisol (TBH) were used as standard whereas dimethyl sulfoxide (DMSO) was used as negative control. Every determination was performed in triplicate. Percentage inhibition of the radical scavenging activity of test samples was calculated according to [19].

(1) Percent  %  inhibition  of  DPPH  activity=Ab−AaAb×100,

where Aa is absorbance values of the test sample, while Ab is absorbance value of blank sample. Extracts concentration providing 50% inhibitions (IC_50_) was calculated from the graph plotted of inhibition percentage against extracts concentration. All tests were carried out in triplicate.

### 2.6. Antifungal Assay (Pour Plate Method)

Antifungal activities of plants crude extract and fraction were determined by pour plate method [20]. Media were prepared by dissolving 65 g of sabouraud dextrose agar (SDA) media in 1000 ml of distilled water. Prepared media were autoclaved for 20 min at 121°C and Petri plates and tips were also autoclaved. Two strains of fungus were used such as* Aspergillus niger* and* Aspergillus flavus* and sample was prepared by dissolving 1 mg/ml in DMSO while 25 ml of media was poured in each Petri plate. Media were allowed to solidify and spores of fungus were applied in the center of the plate. Sterile paper discs were placed in front of the growing end of the mycelium. Samples were applied on the sterile paper discs; each sample was applied in triplicate. The culture was allowed to grow for four to seven days at 26°C. Fluconazole was used as standard and (DMSO) as negative control. Crescent shape of fungi appeared in front of paper discs, showing an inhibition. Zone of inhibition was measured according to [21].

(2) %  Inhibition  of  fungal  growth=100−linear  growth  in  test  (mm)linear  growth  in  control  mm×100.

### 2.7. Antibacterial Assay (Disc Diffusion Method)

Antibacterial activities of the plants crude extracts and different fraction were determined by using paper disc diffusion method [20]. Media were prepared by dissolving 28 g of nutrient agar and 13 g of nutrient broth in 1000 ml of distilled water in flask. The nutrient broth was taken approximately 7-8 ml per test tube. All apparatus and media, namely, Petri plates, tips, Whitman filter paper disc, normal saline, and so on used in activity, were autoclaved for 20 min at 121°C. After sterilization nutrient agars were poured into the Petri plates in laminar flow hood, allowed to solidify, and placed in the incubator at 37°C to avoid any contamination. The bacterial stock cultures were freshened on the nutrient agar plates by streaking with sterile inoculation loop in laminar flow hood; cultures were incubated at 37°C for 24 hours. The bacterial cultures were inoculated into the sterilized nutrient broth in the flask containing approximately 20–25 ml broth media and were, then, incubated in the shaking water bath (Model GLSC-SBR-04-28) for 16 hours at 200 rpm at 37°C. The bacterial cultures from flask were diluted in test tube containing sterilized nutrient broth for standardization by comparing with 0.5 McFarland (turbidity) standards. 50 *µ*l of standardized bacterial culture was spread on each nutrient ager plate with the help of glass spreader. These impregnated plates were refrigerated for 10 mints for absorption, after refrigeration impregnated plates were brought to laminar flow to place the filter paper disc (6 mm in diameter) with the help of sterilized forceps. The samples (6 *µ*ml) were applied on each paper disc. Azithromycin and tetracycline were applied on separate plates as positive control for both bacteria while DMSO was used as negative control. The plates were incubated at 37°C for 24 hours; zone of inhibition was recorded around each paper disc in mm. All tests were applied in triplicate.

### 2.8. Statistical Analysis

All experimental data of antioxidants, antifungal, and antibacterial activities were statistically express as mean ± standard deviation (SD). The measurements are replicated three times. The IC_50_ values for antioxidants were calculated from linear regression analysis using Graph pad prism5 software.

## 3. Results and Discussion

### 3.1. Antioxidant Activities

The crude extract and their fraction of root and stem of* E. gerardiana* were investigated for free radical scavenging activities. The IC_50_ values with 2.96 ± 0.39 and 6.38 ± 1.59 *µ*g/ml were observed in ethyl acetate and chloroform fractions of root, being considered as strong free radical scavenging potentials. Whereas the IC_50_ values of methanolic extract *n*-butanol and n-hexane fractions were 14.94 ± 3.54, 13.74 ± 2.71, and 21.49 ± 6.26, respectively, with weak antioxidant activities (Table 1); the aqueous fraction was inactive in initial screening (data not shown).

Furthermore, the stem crude extract and fractions with the IC_50_ values 3.44 ± 0.69, 2.73 ± 0.84, and 2.69 ± 0.26 *µ*g/ml were documented in aqueous, ethyl acetate, and *n*-butanol fractions, while methanol extract was inactive (data not shown). However, the *n*-hexane and chloroform fractions of stem showed IC_50_ values of 13.92 ± 6.04 and 22.73 ± 6.92 *µ*g/ml (Table 2). In comparison, both root and stem almost have same antioxidative potential. The growing interest in the replacement of synthetic food antioxidants with natural ones has been fostered research on plant sources and screening of raw materials to identify new antioxidants [22, 23]. On the other hand, the antioxidant activities of the methanol extract of* E. gerardiana* (leaf) with IC_50_ value 13.30 ± 0.6 *µ*g/ml showed highest free radical scavenging activities [14], which is in line with the present results; the methanol extracts of* E. gerardiana* (root and stem) showed antioxidant activities with IC_50_ values 14.94 ± 3.54 and 22.73 ± 6.92 *µ*g/ml, respectively (Tables 1 and 2); the difference may be because these parts contain different percentage of bioactive compounds. Hence, other ephedra species such as* E. laristanica* and* E. sarcocarpa* have been studied for natural antioxidant compounds [2, 13]. In light of the these results comparatively overall aqueous, ethyl acetate, and* n*-butanol fractions of stem of* E. gerardiana* might be used as strong antioxidative drug while chloroform fractions of root are also the most applied source of antioxidant.

### 3.2. Antifungal Activity

Based on strong antioxidant potentials, the methanolic crud extract and their fraction were evaluated for antifungal activities against* A. niger* and* A. flavus*. Hence, interesting phenomena have been reported; neither crude extract nor any fraction showed activities against used fungal strains as shown in Table 3. The chemical crude extracts of various medicinal plants other than* E. gerardiana* showed significant antifungal activity against aspergillus species [24, 25], while present chemicals crude extracts inactive against aspergillus species were observed in the present investigation. Similarly, methanol extract of* E. procera* wild plant significantly showed highest antifungal activity against* Candida albicans* [4] and acetonitrile extracts of* E. alata* exhibited the most potent effects against* Aspergillus fumigatus* [26].

### 3.3. Antibacterial Activity

**Figure 2:**
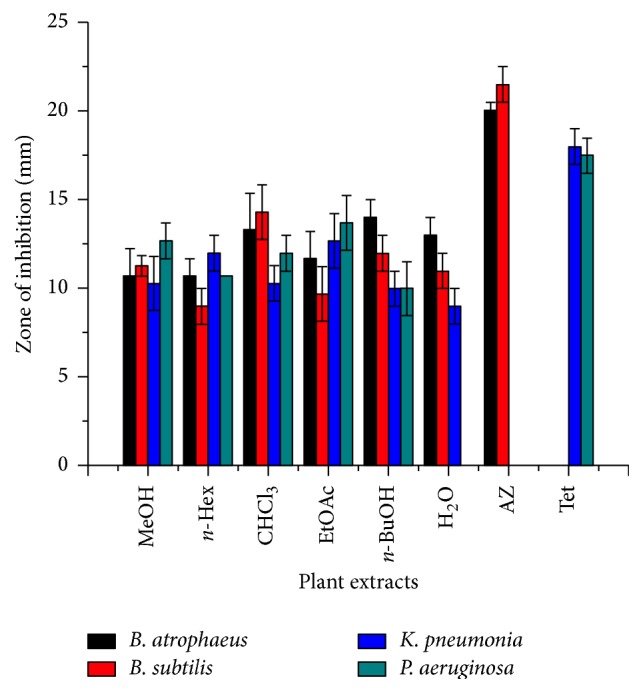
Zone of inhibition of crude methanolic extract and their fractions of* E. gerardiana* (root) of bacterial strains.

In order to further investigate the crude extracts and fractions for antibacterial activities, the root extract showed prominent zone of inhibition against the gram positive and gram negative bacterial strains. The highest zones of inhibition (mm) 13.3 ± 2.08 and 14.3 ± 1.53 mm were documented in chloroform fraction against* B. atrophaeus* and* B. subtilis* although *n*-Butanol fraction also showed 14 ± 1 and 12 ± 1 mm zone of inhibition (mm) against the same strains. Similarly ethyl acetate fraction with 12.7 ± 1.53 and 13.7 ± 1.53 mm zone of inhibition (mm) against* K. pneumonia* and* P. aeruginosa* strain revealed 11.7 ± 1.53 and 9.7 ± 1.53 mm zone of inhibition against* B. atrophaeus* and* B. subtilis* bacterial strains. The aqueous fraction showed 13 ± 1 mm zone of inhibition against* B. atrophaeus* and 9 ± 1 mm against* K. pneumonia* while the same fraction showed no activity against* P. aeruginosa*. Comparatively, the methanolic extract showed average activities against both gram positive and gram negative bacterial strains as compared to the other fractions (Figure 2).

**Figure 3:**
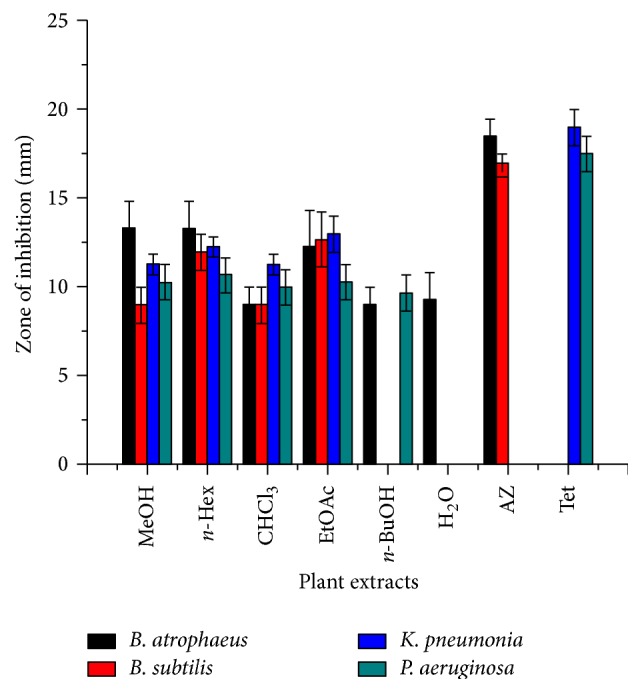
Zone of inhibition of crude methanolic extract and their fractions of* E. gerardiana* (stem) of bacterial strains. Key: MeOH: methanol extract; *n*-Hex: *n*-Hexane fraction; CHCl_3_: chloroform fraction; EtOAc: ethyl acetate fraction; *n*-BuOH: *n*-butanol fraction; H_2_O: aqueous fraction; Az: Azithromycin; and Tet: tetracycline).

On the other hand, methanolic crude extract of stem showed 13.3 ± 1.53 zone of inhibition against* B. atrophaeus,* whereas, *n*-hexane fraction showed the same zone of inhibition against the* B*.* atrophaeus* but 12 ± 1, 12.3 ± 0.58 mm against* B. atrophaeus* and* K. pneumonia*. Ethyl acetate fraction was more active throughout all fractions against both gram positive and gram negative bacteria 13 ± 1, 12.3 ± 2.08, 12.7 ± 1.53, and 10.3 ± 1 mm. The *n*-butanol fraction showed the activity 9 ± 1 mm against* B. atrophaeus* and showed 9.7 ± 1 mm zone of inhibition against* P. aeruginosa* but no activities were observed against* B. subtilis* and* K. pneumonia.* Similarly aqueous extracts showed 9.3 ± 0.58 mm zone of inhibition against* B. atrophaeus* while they were inactive against the other three bacterial strains, that is,* B. subtilis*,* K. pneumonia,* and* P. aeruginosa* (Figure 3). Plants of the genus ephedra have traditionally been used by indigenous people for a variety of medicinal purposes, including treatment of asthma, hay fever, and common cold [27]. The methanol extract of whole plant* E. gerardiana* exhibited maximum antibacterial activity against* Escherichia coli* (2.57 mm) at 15 mg/ml compared with the other two bacterial strains such as* Staphylococcus aureus* and* P. aeruginosa* [12]. In comparison, the* E. gerardiana* (root and stem) showed the highest antibacterial activity against most of chosen microorganisms; in regard to our test it seems that the plant chloroform fraction of root significantly revealed antibacterial potential against* B. atrophaeus* and* B. subtilis* (Figure 2). In contrast, the aqueous fraction of stem was found to be inactive against* B. subtilis*,* K. pneumonia*, and* P. aeruginosa* (Figure 3) as compared to the ethanol extract of* E. gerardiana* (leaf) which also showed prominent zone of inhibition against* E. coli*,* S. aureus*, and* P. aeruginosa* while being inactive against* Bacillus subtilis* [14]. The current data showed that the root and stem crude extracts of* E. gerardiana* contain antibacterial compounds.

## 4. Conclusion

Biological active compound from plant material is mostly dependent on the type of the solvent used in the extraction procedure; in the present study* E. gerardiana* extract and fraction possess antioxidant and antibacterials activities; this might be helpful in preventing or slowing the progress of various oxidative stresses and against pathogenic bacterial strains. Thus, they could be potential candidates for pharmaceutical drugs industries. Further investigation on the isolation and identification of antioxidant and antibacterial component in the plant may lead to chemical entities with potential for clinical use.

## Figures and Tables

**Table 1:** Antioxidant activities of crude methanolic extract and fractions of *E. gerardiana* (root).

Extract/fraction	IC_50_ ± STD *µ*g/ml
Methanolic extract	14.94 ± 3.54
*n*-hexane fraction	21.49 ± 6.26
Chloroform fraction	6.38 ± 1.59
Ethyl acetate fraction	2.96 ± 0.39
*n*-Butanol fraction	13.74 ± 2.71
3-Tert-butyl-4-hydroxyanisol (TBH)	1.2 ± 0.1

**Table 2:** Antioxidant activities of *E. gerardiana *(stem) fractions.

Extract/fraction	IC_50_ ± STD *µ*g/ml
*n*-Hexane fraction	13.92 ± 6.04
Chloroform fraction	22.73 ± 6.92
Ethyl acetate fraction	2.73 ± 0.84
*n*-Butanol fraction	2.69 ± 0.26
Aqueous fraction	3.44 ± 0.69
Propyl gallate	1.6 ± 0.05

**Table 3:** Antifungal activities of *E. gerardiana* (root and stem) of crude methanolic extract and fractions.

Extract/fraction	*A. flavus*	*A. niger*
Methanolic extract	—	—
*n*-Hexane fraction	—	—
Chloroform fraction	—	—
Ethyl acetate fraction	—	—
*n*-Butanol fraction	—	—
Fluconazole	21.4 ± 0.5	29 ± 0.0
